# Antiproliferative and Antioxidant Activities and Mycosporine-Like Amino Acid Profiles of Wild-Harvested and Cultivated Edible Canadian Marine Red Macroalgae

**DOI:** 10.3390/molecules21010119

**Published:** 2016-01-21

**Authors:** Yasantha Athukorala, Susan Trang, Carmen Kwok, Yvonne V. Yuan

**Affiliations:** School of Nutrition, Ryerson University, 350 Victoria St., Toronto, ON M5B 2K3, Canada; yasantha7@yahoo.com (Y.A.); susan.trang1@gmail.com (S.T.); wckwok@ryerson.ca (C.K.)

**Keywords:** antioxidant activity, antiproliferative activity, apoptosis, *Chondrus crispus*, *Mastocarpus stellatus*, *Palmaria palmata*, mycosporine-like amino acids (MAAs), caspase 3/7, HeLa cells, U-937 cells

## Abstract

Antiproliferative and antioxidant activities and mycosporine-like amino acid (MAA) profiles of methanol extracts from edible wild-harvested (*Chondrus crispus*, *Mastocarpus stellatus*, *Palmaria palmata*) and cultivated (*C. crispus*) marine red macroalgae were studied herein. Palythine, asterina-330, shinorine, palythinol, porphyra-334 and usujirene MAAs were identified in the macroalgal extracts by LC/MS/MS. Extract reducing activity rankings were (*p* < 0.001): wild *P. palmata* > cultivated *C. crispus* = wild *M. stellatus* > wild low-UV *C. crispus* > wild high-UV *C. crispus*; whereas oxygen radical absorbance capacities were (*p* < 0.001): wild *M. stellatus* > wild *P. palmata* > cultivated *C. crispus* > wild low-UV *C. crispus* > wild high-UV *C. crispus*. Extracts were antiproliferative against HeLa and U-937 cells (*p* < 0.001) from 0.125–4 mg/mL, 24 h. Wild *P. palmata* and cultivated *C. crispus* extracts increased (*p* < 0.001) HeLa caspase-3/7 activities and the proportion of cells arrested at Sub G_1_ (apoptotic) compared to wild-harvested *C. crispus* and *M. stellatus* extracts. HeLa cells incubated with wild *P. palmata* and cultivated *C. crispus* extracts also exhibited morphological changes characteristic of apoptosis (shrinkage, rounding). Thus, extracts rich in low-polarity usujirene and polar palythine and asterina-330 MAAs were antiproliferative as inducers of apoptosis in HeLa cells.

## 1. Introduction

Marine macroalgae, or seaweeds (sometimes referred to as sea vegetables) play an integral role in the traditional diets of Pacific and Asian peoples, for reasons associated with the macro- (e.g., protein, lipid, fiber) and micronutrient (e.g., iodine, iron, potassium, β-carotene, tocols *etc.*) contents, flavor and texture enhancing properties (e.g., alginates, fucans, agar, carrageenans *etc.*) of the various marine macroalgae and constituents, as well as potentially contributing to a reduction in diet-related chronic disease risk (*i.e*., breast and colorectal cancers) in these populations [1]. Marine macroalgae are also present, but much less ubiquitous, in the traditional diets of Icelandic, Welsh, Irish, North, Central and South American coastal peoples. Amongst these edible macroalgae are many species from the Rhodophyta such as “Nori” or “Kim/Gim” (*Pyropia tenera* or *P. yezoensis*) in Japan and Korea, “Hana Tsunomata™” (cultivated *Chondrus crispus*) for the Japanese market, “Ceylon moss” (*Gracilaria* *edulis*) in South-west and –east Asia, “Dulse” or “Dillisk/Dilleasc/Creathnach” (*Palmaria palmata*) in Ireland, Scotland, Iceland, Norway, Atlantic Canada and U.S.A. While many of these red macroalgae are sun- or shade-dried, then toasted/roasted or rehydrated and consumed whole such as Nori, Dulse and Hana Tsunomata™; others are utilized as sources of hydrocolloid ingredients for the food industry, such as the carrageenophytes consisting of many members of the order Gigartinales, including “Irish moss” (*Chondrus crispus*), “False Irish moss” (*Mastocarpus stellatus*), *Eucheuma* sp., *Kappaphycus* sp. and “Red String seaweed” (*Sarcodiotheca gaudichaudii*); or even direct aqueous extraction of *C. crispus* for use in regional cuisines such as the Seaweed Pie in Prince Edward Island, Canada or Irish Moss drinks in Central America and the Caribbean.

The first functional food or nutraceutical applications for edible macroalgae date back to approx. 1534 B.C. Egypt, where they were used for the treatment or prevention of breast cancer [1], which has been more recently substantiated by a case-control study where intake of Gim was inversely related to breast cancer risk [2]. Epidemiological data support the hypothesis of diet-related chronic disease risk reduction in populations known to consume macroalgae regularly in the diet: the age-standardized incidence of breast cancer in North America and Western Europe are approx. 76.7 and 89.9 per 100,000 compared to 25.3 and 31.0 in Eastern and Southeastern Asia [3]. Similarly, the incidence of colorectal cancer in North America (35.3 and 25.7 per 100,000 for males and females) and Western Europe (41.2 and 26.3) are greater than in Eastern (21.5 and 14.8) and Southeastern Asia (15.2 and 12.9). Anticarcinogenic mechanisms contributing to the bioactivities of edible macroalgae include increased hepatic antioxidant enzyme activities [4]; radio- and photo-protective effects of algal extracts against UVB (280–320 nm; [5]), UVA (320–400 nm; [6]) and ionizing sources of radiation [4]; as well as induction of apoptosis and cell cycle arrest [7]. Moreover, anti-oxidant and/or -mutagenic effects of edible macroalgae were reported from *in vitro* and rodent models of colon and skin carcinogenesis, respectively, including tumour initiation suppression in the latter [8,9]. *Porphyra umbilicalis* extracts protected fibroblasts and keratinocytes against DNA damage and UVA irradiation as described by Schmid and coworkers [10,11]. Thus, there is growing interest in the nutraceutical, pharmacognosic and cosmeceutical applications of extracts from edible marine red macroalgae such as *C. crispus* which is a formulation component contained in an international patent application for anti-aging skin cream uses [12].

**Figure 1:**
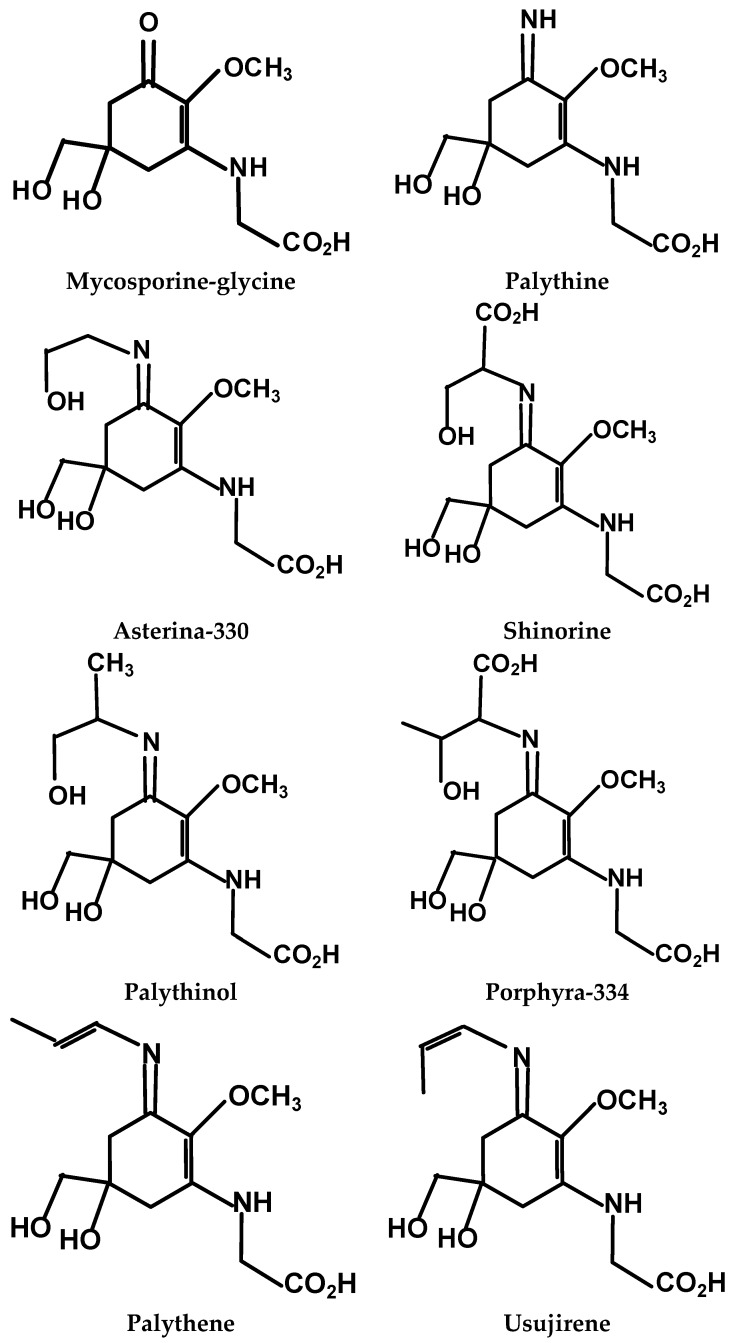
The main mycosporine-like amino acids (MAAs) identified in Rhodophyta.

Many of the antioxidant and UV-protective bioactivities of edible red macroalgae and extracts above can be attributed to the efficacy of cellular constituents against oxidative stress resulting from exposure to temperature variances, tidal flows and UV-irradiation of these intertidal species [13,14,15,16]. For example, macroalgal tissue antioxidants (e.g., ascorbate and carotenoids) are typically reduced during Winter and Spring, and increase in Summer and Fall in concert with increased photosynthetically active radiation (PAR, 400–700 nm) and UVA and UVB-irradiation [14,17,18]. Mycosporine-like amino acids (MAAs), comprised of an aminocyclo-hexenone or -hexenimine core conjugated with the nitrogen moiety of an amino-acid or –alcohol (Figure 1), are most abundant in Rhodophyta (red) compared to Chlorophyta (green) and Pheophyta (brown) macroalgal species [19,20,21,22]. MAAs are noted to exhibit λ_max_ between 310–360 nm, thus, an UV-absorbing sunscreen protective role for these compounds in macroalgae, corals and marine animals can be deduced from the overlap with UVA and B wavelengths, as well as evidence that the MAA contents of *P. palmata* and *Devaleraea ramentacea* specimens wild-harvested prior to the break-up of Spring ice cover were decreased compared to counterparts harvested during the Summer [17]. Previous work from this laboratory demonstrated that *P. palmata* specimens wild-harvested from areas differing in topography exhibited varied MAA profiles: extracts from both low- and high-UV exposed *P. palmata* contained palythine, shinorine, asterina-330, palythinol and porphyra-334, but the high-UV specimen alone, also contained usujirene [13]. Interestingly, despite similar oxygen radical absorbance capacity (ORAC) antioxidant activities between the two *P. palmata* extracts, that from the high-UV specimen was more inhibitory of B16-F1 murine skin melanoma cell proliferation. Previously, butanol extracts of wild-harvested high-UV exposed *P. palmata* exhibited enhanced reducing activity and inhibition of HeLa cell proliferation *vs.* a low-UV exposed counterpart [14].

Clearly then, as photosynthetic, intertidal organisms, the composition of macroalgae within the same species may be highly variable when wild-harvested; thus, it is important that mariculture researchers and producers are cultivating macroalgae from uniform seed stocks in tanks with filtered seawater, fertilizer or other essential nutrients as well as controlled illumination [12,23,24]. Management techniques such as these ensure not only a reliable, year-round supply of biomass with desired attributes such as the Hana Tsunomata™ produced by Acadian Seaplants, Nova Scotia, Canada, but also potentially give rise to genetic variants of interest from target species. A large body of knowledge exists of the MAA composition of wild-harvested red marine macroalgae from around the globe [19,21,22,25,26,27]; however, much less is known about Canadian and North American specimens [13] and in particular, cultivated macroalgae [23]. Thus, the objectives of the present study were to determine the MAA profiles and antioxidant activities of extracts from selected wild-harvested and cultivated Atlantic Canadian edible marine red macroalgae, and to determine the effects of these extracts on the proliferation of two human cancer cell lines, one adherent: cervical adenocarcinoma, HeLa cells, and the other suspended: histiocytic lymphoma, U-937 cells.

## 2. Results

### 2.1. Red Macroalgal Extract Mycosporine-Like Amino Acid Profiles

Multiple reaction monitoring (MRM) positive ion fragmentation pattern LC/MS/MS data confirmed the presence of six MAAs in the wild-harvested and cultivated red macroalgal methanol extracts (in order of elution): palythine, asterina-330, shinorine, palythinol, porphyra-334 and usujirene (Table 1). Palythine was present in substantial quantities in all five red macroalgal extracts with greatest peak area counts in cultivated *C. crispus* and moderate counts in wild-harvested high-UV *C. crispus*, *P. palmata* and low-UV *C. crispus*, and less in the wild-harvested *M. stellatus* extract. While asterina-330, with greatest peak area counts in cultivated *C. crispus* and wild-harvested high-UV *C. crispus*, and shinorine with greatest peak area counts in wild-harvested *M. stellatus* and cultivated *C. crispus*, were also present in all five extracts, albeit at lower peak area counts (Table 1). Palythinol was present in sizeable quantities in the wild-harvested *P. palmata* extract, with much less in wild-harvested *M. stellatus* and trace amounts in the wild-harvested high-UV *C. crispus* extract, but none detected in the others, based on the decreased confidence of identification when peak area counts range between 2000–3000 [28]. Similarly, porphyra-334 was present in sizeable quantities in only the wild-harvested *P. palmata* extract, with much lower peak area counts in the wild-harvested *M. stellatus*, high-UV and cultivated *C. crispus* extracts and trace amounts in the wild-harvested low-UV *C. crispus* extract. Usujirene was present in substantial quantities in only the wild-harvested *P. palmata* and *M. stellatus* extracts, with none detected in the other extracts (Table 1).

**Table 1:** Mycosporine-like amino acid composition of wild-harvested and cultivated red macroalgal extracts determined from LC/MS/MS multiple reaction monitoring ^1^.

	Palythine	Asterina-330	Shinorine	Palythinol	Porphyra-334	Usujirene
Red Macroalgae			Peak Area Counts (×10^3^)			
Wild *P. palmata*	5040	2040	658	3120	5220	13,000
Wild low-UV *C. crispus*	5010	2720	268	ND ^1^	8.8	ND ^1^
Wild high-UV *C. crispus*	6930	4110	233	7.8	24.9	ND ^1^
Cult. *C. crispus*	19,000	6160	2440	3.1	23.4	ND ^1^
Wild *M. stellatus*	3470	1880	6600	98.6	315	1610

^1^ ND = none detected.

The wild-harvested *P. palmata* extract contained the greatest MRM total peak area counts (29,078 × 10^3^) for MAAs, followed by cultivated *C. crispus* (27,627 × 10^3^), wild-harvested *M. stellatus* (13,974 × 10^3^), high-UV *C. crispus* (11,306 × 10^3^) and low-UV *C. crispus* (8007 × 10^3^; Table 1) extracts. The wild-harvested *P. palmata* extract alone contained substantial peak area counts of all six confirmed MAAs with usujirene predominating (Table 1); while that of wild-harvested *M. stellatus* contained substantial peak area counts for all six MAAs with shinorine predominating, followed by palythine, asterina-330, usujirene and smaller amounts of porphyra-334 and palythinol. The cultivated and wild-harvested high-UV and low-UV *C. crispus* extracts contained substantial peak area counts of three MAAs with palythine predominating (particularly in the cultivated specimen), followed by asterina-330, shinorine and small amounts of porphyra-334 (Table 1). It is noteworthy that the wild-harvested high-UV *C. crispus* extract contained a greater total peak area count for MAAs compared to the low-UV specimen, including greater peak area counts for palythine, asterina-330 and porphyra-334.

### 2.2. Red Macroalgal Extract Antioxidant Activities

The reducing activities of the wild-harvested and cultivated red macroalgal extracts varied in the following order (*p* < 0.001): wild-harvested *P. palmata* > cultivated *C. crispus* = wild-harvested *M. stellatus* > wild-harvested low-UV *C. crispus* > high-UV *C. crispus* (Table 2). On the other hand, ORAC values of the wild-harvested and cultivated red macroalgal extracts varied as follows (*p* < 0.001): wild-harvested *M. stellatus* > *P. palmata* > cultivated *C. crispus* > wild-harvested low-UV *C. crispus* > high-UV *C. crispus* (Table 2).

**Table 2:** Reducing activity and oxygen radical absorbance capacity (ORAC) of extracts from wild-harvested and cultivated red macroalgae.

Red Macroalgae	Reducing Activity (mg Ascorbic Acid Equivalents/g Extract)	ORAC (μmoles Trolox Equivalents/g Extract)
Wild *P. palmata*	3.61 ± 0.171 ^a^	45.13 ± 0.674 ^b^
Wild low-UV *C. crispus*	1.02 ± 0.003 ^c^	21.58 ± 0.46 ^d^
Wild high-UV *C. crispus*	0.789 ± 0.088 ^d^	14.15 ± 0.31 ^e^
Cult. *C. crispus*	2.06 ± 0.030 ^b^	33.02 ± 1.12 ^c^
Wild *M. stellatus*	1.58 ± 0.154 ^b^	57.83 ± 0.722 ^a^

^a−e^ indicate significant differences between red macroalgal extracts (*p* < 0.05) within a column.

### 2.3. Red Macroalgal Extract Antiproliferative Effects on HeLa and U-937 Cell Lines

**Figure 2:**
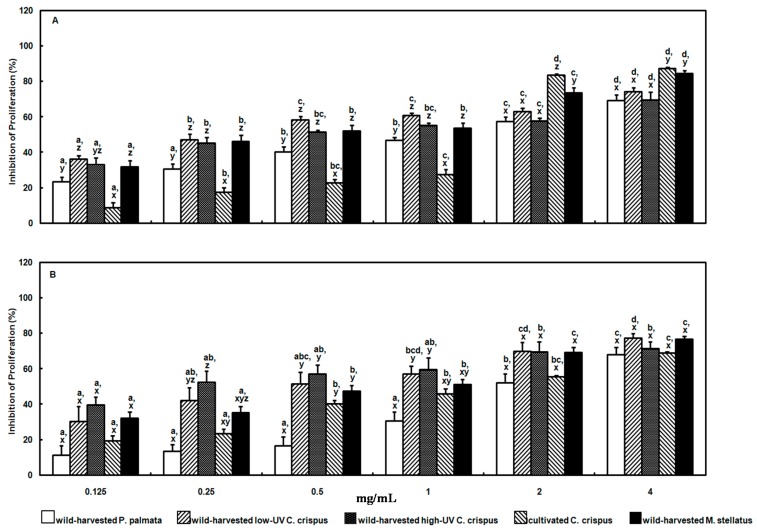
Effect of wild-harvested and cultivated red macroalgal extracts on the proliferation of human cervical adenocarcinoma HeLa (Panel **A**) and histiocytic lymphoma U-937 (Panel **B**) cells over 24 h. ^a–d^ indicate a significant difference (*p* < 0.001) between concentrations of each red macroalgal extract; ^x–z^ indicate a significant difference (*p* < 0.001) between the red macroalgal extracts at each concentration.

The wild-harvested and cultivated red macroalgal extracts inhibited HeLa cell proliferation in a dose-dependent manner (*p* < 0.001) after 24 h incubation (Figure 2A). There were also significant differences (*p* < 0.001) in the inhibition of HeLa cell proliferation between the wild-harvested and cultivated red macroalgal extracts at all concentrations tested. Overall, the strongest antiproliferative effects (*p* < 0.001) were observed with the extracts from the wild-harvested *M. stellatus* = low-UV *C. crispus* > high-UV *C. crispus* > *P. palmata* extract > cultivated *C. crispus* extract. There was an interaction (*p* < 0.001) between dose and red macroalgal extract in that the cultivated *C. crispus* extract exhibited the least efficacy at the low dose concentrations (0.125–1.0 mg/mL), but high efficacy at the highest dose concentrations (2.0–4.0 mg/mL) compared to the other red macroalgal extracts (Figure 2A). Conversely, the wild-harvested low-UV *C. crispus* extract exhibited high efficacy at the lower concentrations (0.125–1.0 mg/mL), but was less efficacious at the highest concentrations (2.0–4.0 mg/mL) compared to the other red macroalgal extracts.

Similar to the HeLa cells above, the wild-harvested and cultivated red macroalgal extracts inhibited U-937 cell proliferation in a dose-dependent manner (*p* < 0.001) after 24 h incubation (Figure 2B). Inhibition of U-937 cell proliferation was significantly different (*p* < 0.001) between the wild-harvested and cultivated red macroalgal extracts at 0.25–1.0 mg/mL concentrations only (Figure 2B). The red macroalgal extract antiproliferative effects (*p* < 0.001) were greatest with the wild-harvested high-UV *C. crispus* ≥ low-UV *C. crispus* ≥ *M. stellatus* ≥ cultivated *C. crispus* > wild-harvested *P. palmata* extracts. While the wild-harvested *P. palmata* extract was the least efficacious compared to the wild-harvested high-UV and low-UV *C. crispus* extracts at the low dose concentrations (0.125–1.0 mg/mL) against U-937 cell proliferation, there were no differences between red macroalgal extract efficacies at the higher dose concentrations (2.0–4.0 mg/mL; Figure 2B). Due to the fewer red macroalgal extract treatment differences observed with U-937 cells above, the remaining cellular analyses to further evaluate the potential mechanisms underlying the antiproliferative effects were performed with HeLa cells only.

### 2.4. Red Macroalgal Extract Effects on HeLa Cell Morphology

**Figure 3:**
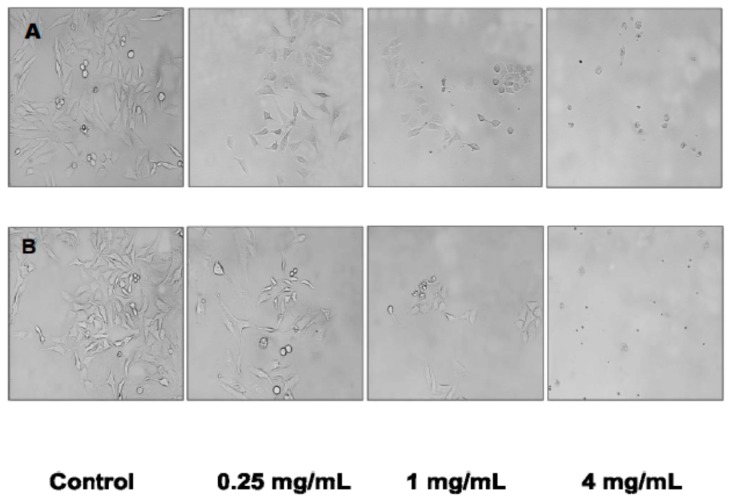
Morphological changes observed in HeLa cells treated with cultivated *C. crispus* (Panel **A**) and wild-harvested *P. palmata* extracts (Panel **B**) using an inverted microscope (200×).

Incubation with the cultivated *C. crispus* (Figure 3A) and wild-harvested *P. palmata* (Figure 3B) extracts induced marked changes in HeLa cell morphology with increasing concentrations of the extracts. HeLa cell morphology was altered from the typical rhomboid–tetrahedral shape observed with the untreated control cells into progressively more contracted and rounded cells characteristic of apoptosis with lower cell density and ultimately, membrane blebbing and apoptotic bodies at the 1.0 and 4.0 mg/mL concentrations.

### 2.5. Red Macroalgal Extract Effects on HeLa Cell Caspase 3/7 Activities

HeLa cell caspase 3/7 activity was influenced by the wild-harvested and cultivated red macroalgal extracts in a dose-dependent manner (*p* < 0.001) after 24 h incubation (Table 3). The greatest induction of caspase 3/7 activity in HeLa cells was observed with 2.00 mg/mL of the wild-harvested *P. palmata* and the cultivated *C. crispus* extracts compared to untreated control cells, all red macroalgal extracts at the lower dose of 0.25 mg/mL and the wild-harvested *M. stellatus* and both high-UV and lo-UV *C. crispus* extracts at 2.00 mg/mL. Thus, only the wild-harvested *P. palmata* and cultivated *C. crispus* extracts were studied for the cell cycle analyses below.

**Table 3:** Effect of wild-harvested and cultivated red macroalgal extracts on HeLa cell Caspase 3/7 activity ^1^.

Red Macroalgae	Extract Concentration	
	0.25 mg/mL	2.00 mg/mL
Wild *P. palmata*	0.98 ± 0.14 ^a^	3.36 ± 0.52 ^c^
Wild low-UV *C. crispus*	0.62 ± 0.10 ^a^	0.91 ± 0.16 ^a^
Wild high-UV *C. crispus*	0.12 ± 0.04 ^a^	0.92 ± 0.06 ^a^
Cult. *C. crispus*	0.16 ± 0.04 ^a^	2.44 ± 0.05 ^b^
Wild *M. stellatus*	0.62 ± 0.32 ^a^	0.68 ± 0.12 ^a^
Control	1.00 ± 0.00 ^a^	

^1^ Caspase (aspartate-specific cysteine proteases) 3/7 activity is expressed as folds over control after 60 min incubation; ^a−c^ indicate significant differences (*p* < 0.05) between doses and red macroalgal extracts.

### 2.6. Red Macroalgal Extract Effects on HeLa Cell Cycle

**Figure 4:**
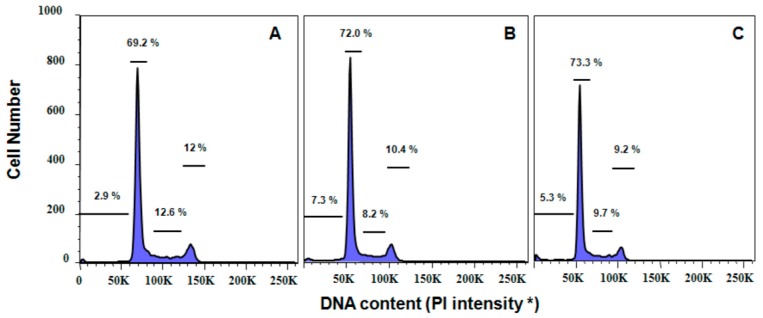
Cell cycle DNA histograms of HeLa cells treated with PBS (Panel **A**); 4 mg/mL cultivated *C. crispus* extract (Panel **B**); and 4 mg/mL wild-harvested *P. palmata* extract (Panel **C**) at 24 h. Values represent (from left to right) the percentages of cells in G_o_, G_1_, S and G_2_M phases. * PI intensity, Propidium iodide in arbitrary units of fluorescence intensity.

The proportions of HeLa cells in the cell cycle phases were also influenced by the wild-harvested *P. palmata* and cultivated *C. crispus* extracts in a dose-dependent manner (*p* < 0.001) after 24 h incubation (Table 4; Figure 4). While the cultivated *C. crispus* extract increased the proportion of HeLa cells in Sub G_1_ from 0.25 to 4.00 mg/mL concentrations and in comparison to untreated control cells, there was only a trend towards an increased proportion of HeLa cells in Sub G_1_ with exposure to the wild-harvested *P. palmata* extract (*p* = 0.155). The wild-harvested *P. palmata* and cultivated *C. crispus* extracts did not influence (*p* > 0.05) the proportions of HeLa cells in the mitosis (M), Gap 1 (G_1_), synthesis (S) or Gap 2 (G_2_) phases (Figure 4).

**Table 4:** Effect of wild-harvested and cultivated red macroalgal extracts on HeLa cell cycle ^1^.

Red Macroalgae	Concentration (mg/mL)	Sub G_1_ (%)
Wild *P. palmata*	0.25	2.89 ± 0.38 ^a^
	2.00	3.43 ± 0.38 ^a^
	4.00	4.13 ± 0.64 ^a^
Cult. *C. crispus*	0.25	3.04 ± 0.33 ^a^
	2.00	5.74 ± 1.24 ^a,b^
	4.00	7.50 ± 1.45 ^b^
Control	0.00	2.69 ± 0.23 ^a^

^1^ 10,000 events were collected from each sample for the analyses; cell cycle phases were analyzed using FlowJo software. ^a,b^ indicate significant differences (*p* < 0.05) between doses and red macroalgal extracts.

## 3. Discussion

This study is the first to report the MAA profiles of aqueous methanolic extracts from a cultivated *C. crispus* specimen as well as wild-harvested *C. crispus* specimens from low- and high-UV exposed locations, wild-harvested *M. stellatus* which co-occurs with *C. crispus* in the intertidal zone and *P. palmata*, all edible red marine macroalgae from temperate zone locations in western Nova Scotia, and Wood and Grand Manan Islands, New Brunswick, Canada, respectively. We extend these findings by reporting the antioxidant capacity, comprising reducing activities and ORAC values of these extracts; as well as the HeLa (cervical) and U-937 (lymphocyte) human adenocarcinoma and histiocytic lymphoma cell antiproliferative activities of these red macroalgal extracts. Moreover, we describe the apoptotic effects of the cultivated *C. crispus* and wild-harvested *P. palmata* extacts on HeLa cells herein. We have previously reported that aqueous methanolic extracts of wild-harvested *P. palmata* specimens from low-UV and high-UV exposed locations on Grand Manan Island (44°40.0’N, 66°45.0’W) exhibited different MAA profiles, with the low-UV sample containing five of the six MAAs herein, except for the low polarity usujirene, and the high-UV *P. palmata* extract containing all six MAAs; however the ORAC values for the extracts were not different [13]. It is noteworthy that the high-UV *P. palmata* extract was more antiproliferative against B16-F1 murine skin melanoma cells over 24-48 h compared to the low-UV extract. Using HPLC-DAD chromatography, we determined that the wild-harvested low- and high-UV *P. palmata* extracts both contained a majority of porphyra-334 and palythine, with smaller amounts of palythinol, shinorine and asterina-330. However, the amount of usujirene in the high-UV *P. palmata* extract was likely underestimated due to the greater λ_max_ (356 nm) and molar extinction coefficient associated with the conjugated double-bond structure of this MAA (Figure 1; [13]). Thus, it is noteworthy that in the present study, LC/MS/MS multiple reaction monitoring indicated that usujirene was the predominant MAA in the wild-harvested *P. palmata* specimen from the same high-UV location, with lower amounts of porphyra-334 and palythine, palythinol, asterina-330 and shinorine. These results with wild-harvested *P. palmata* specimens from a temperature zone climate, are different from those of *P. palmata* harvested from Arctic waters (Spitsbergen, Norway; 78°55.5’N, 11°56.0’E) containing a majority of porphyra-334, with smaller amounts of a compound with λ_max_ of 357 nm (possibly usujirene) and palythine, palythene (trans isomer of usujirene; Figure 1), mycosporine-glycine and palythinol [21]. Compared to other MAAs, usujirene is noted to absorb strongly in the UVA wavelength range which predominates at lower latitudes, in combination with stronger solar radiation, a shorter light path and thinner ozone layer [13,20]. Furthermore, it is noteworthy that porphyra-334 may undergo conversion to usujirene via the shikimic acid pathway [29].

To the best of our knowledge, the present study is the first to elucidate the MAA profile of a cultivated specimen of *C. crispus*. The carrageenophytes *C. crispus* and *M. stellatus* are not only similar in morphology, but also co-occur at the shoreline with *C. crispus* inhabiting the low intertidal zone and *M. stellatus* higher up on shore and in a mixed zone. Interestingly, these two species of red macroalgae (from two different families within the order Gigartinales) vary considerably in MAA synthesis and thereby, profiles: *C. crispus* is noted for a high rate of synthesis of shinorine under UV-irradiation, and this MAA is amongst the first to be synthesized by this species [20,30]. Exposing *C. crispus* to PAR + UV-irradiation results in the additional synthesis of palythine: when *C. crispus* (containing traces of palythine; collected from the North Sea, 54°11’N, 7°53’E) was exposed to unfiltered light as well as light with UVA and UVB filters, shinorine was rapidly synthesized followed by a decline in this MAA, and induction of palythine and asterina-330 [31]. When *C. crispus* (containing palythine, asterina-330 and palythene) was exposed to UVB irradiation, shinorine was synthesized *de novo* and levels of asterina-330 and palythene increased two-fold, while the amount of palythine remained unchanged [30]. Thus, the predominance of palythine , followed by asterina-330 and lesser amounts of shinorine in the wild-harvested low- and high-UV *C. crispus* extracts (from western Nova Scotia (43°82’N, 66°15’W) herein, are in agreement with an inverse relationship (*i.e*., reciprocal changes reflecting a precursor-product relationship) between shinorine and palythine, as well as asterina-330 concentrations in this red macroalga [20]. The greater MAA total peak area counts for the wild-harvested high-UV *C. crispus* extract compared to the low-UV specimen are indicative of the increased oxidative stress, solar and UV-irradiation exposure associated with the shallow water habitat of the former, compared to the deeper water location of the latter. Moreover, the increased amounts of the most abundant MAAs palythine (λ_max_ 320 nm), asterina-330 (λ_max_ 330 nm) as well as minor amounts of porphyra-334 (λ_max_ 332 nm) and palythinol (λ_max_ 330 nm) but similar amounts of shinorine (λ_max_ 332) in the wild-harvested high-UV *C. crispus* extract reflect the greater exposure to UVA-irradiation in the temperate zone shallow water depths of western Nova Scotia and thereby bioconversion of shinorine to form palythine and asterina-330 as above. It is noteworthy that the cultivated *C. crispus* extract contained 2.44 × the MAA total peak area counts compared to the wild-harvested high-UV specimen, reflecting a greatly increased synthesis of shinorine and bioconversion of this MAA to yield palythine and asterina-330. The cultivated *C. crispus* was very highly pigmented, appearing uniformly dark purple compared to the variegated red purple with some green, and pale green of the wild-harvested low-UV and high-UV *C. crispus*, respectively. These pigmentation differences reflect the tissue levels of the key light-harvesting pigment in red macroalgae, (*R*)-phycoerythrin, which is known to vary inversely with UV-irradiation exposure [14]. Thus, the cultivation techniques used have uniquely maximized MAA synthesis of the selected *C. crispus* seedstock in tanks with micro-filtered seawater and nutrients [32]. Interestingly, *M. stellatus* extracts are often comprised solely of shinorine [21,25,30,33]. For example, *M. stellatus* (wild-harvested from the North Sea, as above) subjected to UVB-irradiation over 5 days, exhibited a 1.67 × increase in shinorine content without any trace of other MAAs [30]. The extract from wild-harvested *M. stellatus* from Wood Island, New Brunswick (44°37’N, 66°50’W) in the present study, contained not only a majority of shinorine, but also considerable amounts of palythine, asterina-330, usujirene and porphyra-334. Thus, it is likely that the lower latitude of growth of the *M. stellatus* herein, compared to the North Sea, exposed the specimen to increased total solar and UVA-irradiation as discussed above, resulting in bioconversion of accumulated shinorine to form palythine and asterina-330, as well as conversion of porphyra-334 into usujirene since the latter absorbs more strongly in the UVA range than the former MAA [20].

In the present study, the aqueous methanol extracts of the edible wild-harvested and cultivated red macroalgae exhibited variable antioxidant efficacies when expressed as single electron transfer reducing activity and hydrogen atom transfer ORAC values. Overall, the red macroalgal extracts exhibited weak reducing activities with 1 g of extract equivalent to milligrams of l-ascorbic acid. This is not unexpected, as sun-drying and storage of macroalgae such as *P. palmata* are noted to markedly reduce the antioxidant molecule content, including l-ascorbate, α- and β-carotene [14]. Alternatively, ORAC values represent the ability of chain-breaking antioxidants to quench free radicals through hydrogen atom transfer to the carbon or peroxyl radicals from AAPH decomposition and reaction with molecular oxygen. Differences in the antioxidant activities between the wild-harvested red macroalgal extracts are indicative of the endogenous cellular antioxidant capacity required to protect against environmental oxidative stresses associated with differences in intertidal zone habitat and UV-exposure. Previously, aqueous methanolic and 1-butanol soluble extracts from wild-harvested high- and low-UV *P. palmata* specimens exhibited similar ORAC and AAPH-radical quenching activities, despite differing MAA profiles and greater reducing activity in the high-UV *P. palmata* 1-butanol extract compared to a low-UV counterpart [13,14]. That the extract from the wild-harvested *P. palmata* specimen (collected from the low-lying exposed eastern shore of Grand Manan Island with high UV-irradiation) exhibited the greatest reducing and second highest ORAC activities herein may be attributable to the total MAA content, but also to the predominance of usujirene in the MAA profile of this red macroalga. Nakayama and coworkers [23] proposed that the strong antioxidant efficacy of usujirene may be attributable to hydrogen ion abstraction from the cycloheximine ring at C-4, C-6 or the methylene group at C-9 of the glycine residue with resonance stabilization from the conjugated double-bonds of the *cis*-unsaturated chain at C-11 on the double-bonded nitrogen in series with the carbon ring double-bond structure. The wild-harvested *P. palmata* extract herein was also characterized by the greatest amount of porphyra-334; this MAA demonstrated moderate inhibition of AAPH-induced lipid peroxidation when present in a 9.6:1 ratio mixture of porphyra-334:shinorine [34].

The differences in intertidal zone habitats occupied by *M. stellatus* compared to *C. crispus* have been identified as conferring greater stress tolerance and a competitive advantage of the former over the latter, as evidenced by a greater total MAA content as well as endogenous antioxidant capacity comprising l-ascorbate, α-tocopherol, β-carotene and antioxidant enzymes; [16,30]. Likewise, herein we report greater MAA total peak area counts for the wild-harvested *M. stellatus* extract *vs.* the high-UV and low-UV *C. crispus* extracts, respectively, in addition to greater reducing and ORAC activities in the former compared to latter species. The stronger antioxidant efficacies of the wild-harvested *M. stellatus* extract likely reflect the hydrogen ion abstraction mechanisms of the main constituent MAA, shinorine associated with the cycloheximine ring at C-4, C-6 or the methylene group at C-9 of the glycine residue with some resonance stabilization from the carbon ring double-bond structure, in addition to the efficacy of the usujirene content, as above [23]. Moreover, Dunlap and coworkers [35] reported the dose-dependent inhibition of AAPH-induced phosphatidylcholine peroxidation by shinorine. It is also plausible that palythine, which when present in a 27.9:9:4.3:1 ratio of mycosporine-glycine:palythine:palythinol:asterina-330, potentially contributed to the strong inhibition of AAPH-induced peroxidation of these MAAs [34], played a role in the antioxidant effects of the wild-harvested *M. stellatus* extract herein. On the other hand, the weaker antioxidant activities of both the wild-harvested high-UV and low-UV *C. crispus* extracts are indicative of the efficacy of the main constituent MAA, palythine, as above, in addition to asterina-330 which exhibited little inhibition of AAPH-induced peroxidation when present in a 8:1 ratio of asterina-330:palythine [34]. The antioxidant activities of the cultivated *C. crispus* extract likely resulted from the efficacy of the predominant MAA, palythine as a hydrogen atom donor as well as that of shinorine, with resonance stabilization from the carbon ring double-bond structure in the latter MAA, as above. These lines of evidence from two different antioxidant mechanisms, suggest that the reducing and ORAC antioxidant activities of the wild-harvested *P. palmata*, *M. stellatus*, high- and low-UV *C. crispus* and cultivated *C. crispus* red macroalgal extracts can be attributed largely to the hydrogen atom transfer efficacies of usujirene, shinorine and palythine, with a minor contribution from asterina-330.

The aqueous methanolic extracts of the wild-harvested and cultivated red macroalgae herein exhibited dose- and species-dependent antiproliferative effects against human adenocarcinoma cervical (HeLa) and histiocytic lymphoma (U-937) cells *in vitro*. The EC_50_ values calculated for the wild-harvested *P. palmata*, low-UV *C. crispus*, high-UV *C. crispus*, cultivated *C. crispus* and wild-harvested *M. stellatus* extract inhibition of HeLa cell proliferation after 24 h incubation were: 1.83, 0.457, 1.06, 1.73 and 0.747 mg/mL; whereas those for U-937 cells were: 2.51, 0.772, 0.344, 1.99 and 1.15 mg/mL. Previously, we reported that aqueous methanolic extracts from a high-UV exposed *P. palmata* specimen exhibited greater antiproliferative effects against B16-F1 murine melanoma cells over 48 h than a low-UV exposed counterpart [13]. It was hypothesized that these effects may be attributable to the passive uptake of the low-polarity, weakly acidic MAA, usujirene across cell membranes. Similarly, Moo-Puc and co-workers [36] proposed that membrane permeation by low-polarity/lipophilic compounds was responsible for the cytotoxic and antiproliferative effects of Yucatán Rhodophyta extracts such as *Gracilaria cervicornis* which exhibited an EC_50_ value of 0.076 mg/mL against HeLa cells. Other workers have reported a dose-dependent and apparently saturable, active transport of the highly polar, strongly acidic MAA, shinorine across cell membranes to be antiproliferative against human skin carcinoma A431 cells [33]. The above EC_50_ values represent low antiproliferative efficacies in comparison to the U.S. National Cancer Institute standard of an EC_50_ ≤ 30 μg/mL extract on cancer cells, which is not surprising, given the mixture of MAAs and varying concentrations present in our extracts. More recently, a case-control study revealed that the average intake and frequency of Gim consumption was inversely associated with breast cancer risk in pre- as well as post-menopausal women [2]. It is noteworthy that several animal models of carcinogen-induced intestinal adenocarcinoma, as well as implanted sarcoma-180 ascites cell studies have attributed the anticarcinogenic efficacies of dietary red macroalgae in part to the sulfated polysaccharides, but also free radical scavenging and antioxidant small molecules [2,37,38].

In the present study, the proliferation of both HeLa and U-937 cells were considerably inhibited by the wild-harvested *M. stellatus* extract containing the polar, strong acid shinorine, albeit the efficacy of this extract was not as strong in the latter cell line. Active transport of shinorine may also have been a factor in the slightly weaker antiproliferative effects of the cultivated *C. crispus* extract on HeLa and U-937 cells, given the reduced peak area counts for shinorine compared to that in the wild-harvested *M. stellatus* extract. Membrane permeation by the low-polarity, weakly acidic usujirene may also have played a role in the antiproliferative effects of the wild-harvested *M. stellatus* extract above. Similarly, usujirene may have been efficacious in the antiproliferative effects of the wild-harvested *P. palmata* extract against HeLa and U-037 cells. On the other hand, the antiproliferative efficacies of the wild-harvested high-UV and low-UV *C. crispus* extracts against HeLa and U-937 cells are unclear at present, but may potentially be associated with the trans-membrane movement of other polar, weakly acidic MAAs, namely palythine and asterina-330.

In other work, the proliferation of HT-29 colon cancer cells was strongly inhibited by 0.020 mg/mL of a methanolic extract from the Rhodophyta *Symphyocladia latiuscula*, associated with an increased proportion of apoptotic cells [7]. Induction of apoptosis (*i.e*., programmed cell death) in the HT-29 cells was associated with activation of the caspase-3/7 cascade (aspartate-specific cysteine proteases) and poly (ADP-ribose) polymerase (PARP) activation. Apoptosis, also known as type I cell death in mammalian cells, is noted to be characterized by distinct alterations in the nucleus (comprising chromatin condensation and fragmentation), cell shrinkage and blebbing of the plasma membrane and ultimately, formation of apoptotic structures containing nuclear or cytosolic components [39]. The mechanisms underlying apoptosis involve proteolytic activity of a family of caspases which participate in the initiation, execution and regulation of apoptosis, leading to breakdown of the cytoskeleton, disruption of cellular metabolism and fragmentation of nuclear material [39]. Thus, the caspase family is noted to comprise upstream initiators (caspase-8, -9 and -10) which activate the downstream effectors of apoptosis (caspase-3, -6 and -7). Activation of caspases can occur by differing signaling routes: the extrinsic death receptor pathway which plays a role in tissue homeostasis and the immune system, or the intrinsic mitochondrial pathway which responds to a variety of extra- or intracellular insults such as DNA damage; it is this latter pathway which predominates in programmed cell death. Thus, it is noteworthy that both the wild-harvested *P. palmata* and cultivated *C. crispus* extracts increased caspase-3/7 activity in HeLa cells. Caspase-3/7 activation has been reported to be responsible for cleaving cell cycle regulators, including PARP, which induce cell cycle arrest, leading to induction of apoptosis as above [7].

The induction of apoptosis in HeLa cells in the present study was confirmed by the dose-response changes in cell morphology as a result of incubation with the cultivated *C. crispus* and wild-harvested *P. palmata* extracts. Moreover, cell cycle analyses also confirmed that apoptosis played a role in the antiproliferative effects of the cultivated *C. crispus* and wild-harvested *P. palmata* extracts on HeLa cells with pronounced dose-dependent Sub G_1_ phase (apoptotic) arrests after 24 h. Interestingly, while the induction of caspase-3/-7 activity in HeLa cells was stronger with the wild-harvested *P. palmata* extract compared to that from cultivated *C. crispus* and the untreated control cells, the accumulation of Sub G_1_ apoptotic cells was greater in HeLa cells exposed to the latter, compared to the former extract. These differences may relate to the timing and duration of the respective phenomena, as apoptosis is noted to occur very rapidly, typically within hours [39]; thus, caspase-3/-7 activation would be expected to precede mitochondrial and DNA fragmentation effects leading to cell cycle arrest. The lack of dose-dependent effects of the wild-harvested *M. stellatus*, low-UV and high-UV *C. crispus* extracts on HeLa cell caspase-3/-7 activity compared to those discussed above, does not eliminate the possibility that initiator caspase (caspase-8, -9 or -10) activities or other cell cycle regulators may have been affected. It is noteworthy that HeLa caspase-3/-7 activities were increased by the two red macroalgal extracts with the greatest MAA total peak area counts in the cultivated *C. crispus* and the wild-harvested *P. palmata* extracts.

In conclusion, the MAA profiles of aqueous methanol extracts from the edible wild-harvested and cultivated marine red macroalgae studied herein exhibited differences attributable to not only species-specific differences in MAA synthesis and bioconversion, but also UV-exposure in a temperate climate zone. Red macroalgal extract reducing and ORAC activities reflected the differing efficacies of component MAAs as electron and/or hydrogen atom donors and free radical quenchers; as well as the relative amounts of MAAs in individual extracts, with the greatest efficacies observed with the wild-harvested *P. palmata*, rich in usujirene, porphyra-334 and palythine, and wild-harvested *M. stellatus* extracts, rich in shinorine. The HeLa and U-937 cell antiproliferative effects of the wild-harvested *P. palmata*, *M. stellatus* and cultivated *C. crispus* extracts likely reflected passive uptake and active transport of usujirene and shinorine, respectively, across cell membranes as discussed above. The antiproliferative effects of the wild-harvested low-UV and high-UV *C. crispus* extracts remain unclear, but may have been due to transport of other polar MAAs across cell membranes. HeLa cell death via apoptosis was confirmed as the mechanism underlying the effects of the wild-harvested *P. palmata* and cultivated *C. crispus* extracts from not only increased effector caspase-3/-7 activities, but also cell-cycle arrest at Sub G_1_ in treated cells; cell morphologies also indicated characteristic apoptotic changes. Further work with purified MAAs will help to further elucidate the protective antioxidant and antiproliferative mechanisms of these unique UV-absorbing marine red macroalgal constituents. Moreover, animal model studies will determine the potential protective bioactivities of dietary wild-harvested and cultivated red macroalgae studied herein, and their constituents, against diet-related chronic diseases. This work will be instrumental in furthering the development of mariculture producers and processors of edible marine red macroalgae in the processed and functional food industries.

## 4. Experimental Section

### 4.1. Materials and Reagents

Three specimens of *Chondrus crispus* (Class Florideophyceae, Subclass Rhodymeniophycidae, Order Gigartinales, Family Gigartinaceae) were kindly provided by Alan Critchley (Acadian Seaplants Limited, Dartmouth, NS, Canada): the first, wild-harvested from western Nova Scotia in deeper waters (low-UV); the second, wild-harvested from western Nova Scotia in shallow waters (high-UV); the third, a cultivated specimen; as well as a *Mastocarpus stellatus* (Order Gigartinales, Family Phyllophoraceae) specimen wild-harvested from Wood Island, New Brunswick. Certified organic (Organic Crop Improvement Association International, Lincoln, NE, USA) *P. palmata* (dulse; Class Florideophyceae, Subclass Nemaliaphycidae ,Order Palmariales, Family Palmariaceae) was obtained from Atlantic Mariculture Ltd. (Grand Manan, NB, Canada). The dulse was wild-harvested from the low-lying eastern shores of Grand Manan Island with increased exposure to UV radiation as previously described by Yuan *et al.* [13,14]. HeLa and U-937 cells were purchased from American Type Culture Collection (ATCC, Manassas, VA, USA). Solvents (formic acid, methanol, acetonitrile (ACN)) were purchased from Fisher Scientific (Mississauga, ON, Canada) and were all of HPLC grade. l-ascorbic acid, trichloroacetic acid (TCA), 6-hydroxy-2,5,7,8-tetramethylchroman-2-carboxylic acid (Trolox), fluorescein Na-salt and 2,2’-azo-bis(2-amidinopropane) dihydrochloride (AAPH), 3-(4,5-dimethylthiazolyl-2)-2,5-diphenyltetrazolium bromide (MTT) were purchased from Sigma-Aldrich Canada (Oakville, ON, Canada). Dulbecco’s phosphate buffered saline (D-PBS), Dulbecco’s modified Eagle’s medium (DMEM), d-glucose, l-glutamine, sodium pyruvate, heat-inactivated foetal bovine serum (FBS), penicillin/streptomycin and TrypLE Express were purchased from Invitrogen Life Technologies (Burlington, ON, Canada). Water (H_2_O) used as a solvent or in mobile phases was purified using a Millipore Super-Q Water System (Millipore, Bedford, MA, USA). Sample absorbances were read using a Lamba 20 UV/Vis Spectrometer (Perkin-Elmer, Norwalk, CT, USA).

### 4.2. Preparation of Macroalgal Extracts

Lyophilized, sun-dried and comminuted red macroalgal samples were ground to 40 mesh screen size prior to soaking in H_2_O, 4 °C, overnight, followed by methanol extraction and centrifugation (4 °C, 15 min, 3000 rpm) for collection of supernatants as previously described by Yuan and coworkers [13]. Sample material was extracted twice more with methanol and the supernatants pooled and concentrated prior to solubilization in H_2_O and sonication. Extracts were filtered (0.20 μ) into a glass vial with an insert (Chromatographic Specialties Inc., Brockville, ON, Canada) for LC/MS/MS analyses, antioxidant and cell proliferation assays.

### 4.3. LC/MS/MS

HPLC-mass spectral analyses were carried out at the Mass Spectrometry Facility for Small Molecules at The Hospital for Sick Children (Toronto, ON, Canada). Mobile phases were 0.2% formic acid in H_2_O and 0.2% formic acid in ACN, respectively. Chromatography was performed with an Agilent 1100 Series LC (Agilent Technologies, Mississauga, ON, Canada) equipped with an Inertsil ODS-3 column (5 μ, 4.6 × 250 mm; GL Sciences, Inc. USA, Torrance, CA, USA) using a gradient elution starting with 0.2% formic acid in H_2_O and ending with 0.2% formic acid in ACN over 20 min, at a flow rate of 1.0 mL/min, 30 °C.

Triple quadrupole LC/MS/MS (API 4000; Applied Biosystems/MDS Sciex Instruments, Toronto, ON, Canada) in positive ion mode with a turbo spray ionization source was used to determine molecular masses of separated MAAs using Analyst NT version 1.4.1 Software. The instrument was operated in the multiple reaction monitoring (MRM) mode to acquire total product ion counts. The transitions monitored ranged between *m*/*z* 347.2 and 186.2 *m*/*z* using nitrogen as the collision gas at 150.9 bar. The declustering potential was −50.00 V, collision energy was −42.00 V, dwell time was 30.0 ms, ion spray voltage (Sciex Turbo Ion Spray TIS Probe) was set to −4200 V and the source temperature was 650.0 °C. MAAs were identified using published data for formula weights, [M + H]^+^ values and fragmentation patterns [13,25,40] in the absence of commercially available MAA standards.

### 4.4. Reducing Activity

The reducing activities of the red macroalgal extracts were determined as previously described by Yuan and coworkers [14]. Sample absorbances were read at 700 nm, and reducing activities expressed as ascorbic acid equivalents from the l-ascorbic acid calibration curve. Each macroalgal extract was assayed in quadruplicate.

### 4.5. Oxygen Radical Absorbance Capacity (ORAC-fluorescein) Assay

The ORAC values of the red macroalgal extracts were determined as described by Yuan and coworkers [13] with modifications. Briefly, aliquots of red macroalgal extracts or the Trolox reference antioxidant were mixed with 60 nM fluorescein in a black 96-well cliniplate (Thermo Scientific, VWR Canlab, Mississauga, ON, Canada) in the presence of 75 mM phosphate buffer, pH 7.0 and incubated at 37 °C, 10 min. AAPH was then added for a final concentration of 12 mM in each well and the plate shaken for 10 s prior to the initiation of fluorescence readings, with excitation and emission wavelengths of 485 and 538 nm, respectively (Fluorskan Ascent, Thermo Scientific) at 37 °C. Fluorescence readings were taken every 60 s for 80 min; the microplate was shaken for 10 s prior to each reading. Raw data were exported from the Ascent Software 2.6 to a spreadsheet for calculation of the areas under the fluorescence decay curves. Red macroalgal extract ORAC values were expressed as μmoles Trolox equivalents/g extract.

### 4.6. Cell Proliferation

HeLa cells were maintained in DMEM with 4.5 g/L d-glucose, l-glutamine, and 110 mg/L sodium pyruvate and supplemented with 10% (*v*/*v*) heat-inactivated FBS and 1% (*v*/*v*) penicillin/ streptomycin in a humidified environment with 5% CO_2_, 37 °C. U-937 cells were maintained in DMEM supplemented with 10% (*v*/*v*) FBS and 1% (*v*/*v*) penicillin/streptomycin in an environment as above. HeLa cells were grown up to 60%–80% confluence in 75 cm^2^ tissue culture treated culture flasks and were passaged using D-PBS and TrypLE Express (trypsin containing 1mM of EDTA in D-PBS); whereas U-937 cells were passaged using PBS and TrypLE Express.

### 4.7. MTT Assay

HeLa and U-937 cells were harvested and seeded (180–190 µL) into flat bottom 96-well plates at a density of 2 × 10^4^ cells/mL for the respective experiments. HeLa cells were allowed to attach for 16 h and then treated with either D-PBS (control) or red macroalgal extracts (10–20 µL) at final concentrations of 0.125-4 mg/mL of media [14]; whereas U-937 cells (suspended cells) were treated 15 h after transfer to 96-well plate, as above. After 24 h incubation, 50 µL MTT solubilized in D-PBS (2 mg/mL) was added to each well, with further incubation for 4 h. U-937 cells were centrifuged in plates, 500× *g*, 21 °C. The reagents from each well were carefully removed by vacuum and 150 µL dimethyl sulfoxide was added. The 96-well plates were mixed on a nutating mixer (VWR Canlab) at 25 rpm, 20 min at room temperature to dissolve the formazan crystals, and the absorbances at 570 nm read with a Multiskan Ascent Microplate Reader (Thermo Labsystems, Franklin, MA, USA). The inhibition of HeLa or U-937 cell proliferation by red algal extracts was calculated as follows:

% Inhibition = (Abs570nm control − Abs570nm sample) ÷ Abs570nm control × 100
(1)

A subset of HeLa cells incubated with the cultivated *C. crispus* and wild-harvested *P. palmata* extracts were viewed and photographed using an inverted microscope at 200× magnification.

### 4.8. Propidium Iodide (PI) Staining for Flow Cytometry

HeLa cells were seeded in a 6-well plate (Cellstar, Greiner Bio-One, Monroe, NC, USA) at a density of 2 × 10^5^ cells/mL and allowed to attach overnight at 37 °C, 5% CO_2_ with a fully humidified atmosphere. Cells were treated with either PBS (control) or red macroalgal extracts (0.25, 2 and 4 mg/mL) and incubated for 24 h. Then, floating and trypsinized HeLa cells were collected, centrifuged at 400× *g*, 4 °C, 5 min. Supernatants were aspirated and the cell pellets washed with 1 mL cold PBS, prior to fixing cells with 1 mL 80% ethanol and storage overnight, 4 °C. Ethanol-fixed HeLa cells were washed twice with 1 mL cold PBS, followed by suspension in 500 µL RNAse (2 mg/mL; Ribonuclease A from bovine pancreas, Sigma-Aldrich) in PBS for 5 min at room temperature, and stained with PI (0.1 mg/mL; Calbiochem Corp., La Jolla, CA, USA) in PBS containing 0.6% (*w*/*v*) of NP-40 (Bioshop Canada Inc., Burlington, ON, Canada) for 30 min in the dark. Cells were strained using filter cap tubes to remove clumps (polystyrene round-bottom tube with cell-strainer cap; BD Falcon™, Franklin Lakes, NJ, USA), wrapped in aluminum foil, placed on ice and analysed immediately using a Becton Dickinson LSRII SC Flow Cytometer at 488 nm excitation (detector D, 600 LP and 610/20 BP; BD Biosciences, San Jose, CA, USA) running BD FACSDiva 6.1.2 software (Becton Dickinson BioSciences, San Jose, CA, USA). 10,000 events were collected from each sample for analyses. Cell cycle phases and profiles were analysed using FlowJo software (Tree Star, Inc. Ashland, OR, USA).

### 4.9. Caspase-3/7 Activity Assay

Caspase-3/7 activity was determined using an Apo-One^®^ Homogeneous Caspase-3/7 Assay kit (Promega Corporation, Madison, WI, USA) according to the manufacturer’s directions. Briefly, HeLa cells were seeded in a black 96-well plate (Cellstar, Greiner Bio-One) at a density of 2 × 10^4^ cells/mL and allowed to attach overnight. Cells were then treated with either PBS (control) or red macroalgal extracts (0.25 and 2 mg/mL) and incubated for 24 h. Apo-ONE™ caspase reagent (100 µL; 1:100 fluorescent substrate and lysis buffer) was added to each well, followed by gentle mixing (360 rpm, 30 s) and incubation at 37 °C, 1 h. The fluorescence intensity was measured (excitation, 485 nm; emission, 538 nm) using a Fluorskan Ascent microplate reader. Background fluorescence was determined from the cell culture medium alone and subtracted from all experimental values. The Caspase 3/7 activity was expressed as folds over control after 60 min incubation.

### 4.10. Statistics

All data are expressed as means ± SEM of quadruplicate experiments. One-way analysis of variance (ANOVA; PASW Statistics for Windows 18; SPSS Inc., Chicago, IL, USA) was used to test for differences between red macroalgal species as well as treatment concentrations. Where differences did exist, the source of the differences at a *p* ≤ 0.05 significance level was identified by the Student-Newman-Keuls multiple range test. Two-way analysis of variance (MANOVA, PASW Statistics) was used to test for interactions between red macroalgal species and treatment concentrations. Student’s *t-*test for independent samples was used to test for differences between low- and high-UV *C. crispus* specimens at a significance level of *p* ≤ 0.05 where appropriate (PASW Statistics).
